# The mediating role of neuroception of psychological safety in the relationship between traumatic experiences and aggressive behaviors among inpatients with mental disorders

**DOI:** 10.3389/fpubh.2026.1842451

**Published:** 2026-09-15

**Authors:** Lei Zhang, Zhiyang Lv, Haiying Min, Yanbo Wang

**Affiliations:** Clinical Research Center for Mental Disorders, Shanghai Pudong New Area Mental Health Center, School of Medicine, Tongji University, Shanghai, China

**Keywords:** aggressive behavior, inpatients, mediating role, mental disorders, psychological safety, traumatic experiences

## Abstract

**Background:**

Patient safety is a central concern in the field of mental health. Violent and aggressive behaviors occur frequently in psychiatric wards and often lead to serious consequences. Existing management strategies predominantly focus on physical interventions and symptom treatment, as well as risk assessment, emergency response planning, and coercive measures, while paying insufficient attention to patients’ psychological perceptions and traumatic experiences. The Polyvagal Theory distinguishes neuroception from conscious psychological safety. Neuroception refers to a subconscious process involved in detecting safety and threat, which may provide a theoretical perspective for understanding the association between trauma and defensive behaviors, including aggression. However, current assessment tools in China, such as the Security Questionnaire developed by Cong Zhong et al., mainly assess conscious psychological safety do not capture this preconscious bodily dimension. The Chinese version of the Neuroception of Psychological Safety Scale (NPSS), culturally adapted and validated by our research team, may address this gap. To date, the potential relationships among traumatic experiences, neuroception of psychological safety, and aggressive behavior among hospitalized patients with mental disorders in China remain insufficiently understood.

**Methods:**

This was a cross-sectional study. 216 inpatients were randomly sampled (2023.7–10). Scales including NPSS, CTQ and MOAS were used for data collection. SPSS 26.0 and AMOS 23.0 were applied for statistical analysis and structural equation modeling, with bootstrap testing of indirect effects.

**Results:**

Neuroception of psychological safety score was 3.80 ± 0.56 (highest somatic perception, lower social engagement/compassion); 90.3% had treatment-related trauma. Seven factors were independently associated with neuroception of psychological safety (all *p <* 0.05, adjusted *R^2^* = 0.581). Traumas correlated positively with aggression and negatively with neuroception of psychological safety, which correlated negatively with aggression (all *p <* 0.01). Significant indirect associations through neuroception of psychological safety were observed in both trauma–aggression relationships, accounting for 24.2 and 20.4% of the total associations, respectively.

**Conclusion:**

Inpatients with mental disorders exhibited relatively lower scores on the dimensions of social engagement and compassion. Neuroception of psychological safety may partially account for the observed association between trauma and aggression. Its potential role as a clinically relevant factor warrants further investigation in longitudinal and interventional studies.

## 1 Introduction

Patient safety is defined as the avoidance, prevention, and amelioration of adverse outcomes or injuries stemming from the processes of healthcare (1). Improving patient safety has long been a critical concern in the field of mental health. Patients with mental disorders frequently exhibit aggressive and violent behaviors, which often result in serious consequences. Statistics indicate that aggressive behavior is one of the most frequently reported adverse safety events in psychiatric wards (2), characterized by its hidden nature, significant destructive potential, and limited predictability (3). Current management strategies for addressing violent and aggressive behaviors primarily focus on psychiatric symptom treatment, risk assessment, emergency response planning, and coercive measures (4). These approaches are largely based on attributing patients’ problematic behaviors to psychopathological symptoms and are typically implemented from the perspective of healthcare providers. Consequently, they often overlook the potential relevance of patients’ own psychological perceptions and traumatic experiences to their behaviors. This highlights a gap in current research: the prevention and management of aggressive behaviors in patients with mental disorders primarily focus on physical interventions and symptom management, overlooking the potential mediating role of neuroception-as distinct from general psychological safety- in the association between trauma and aggression. Furthermore, there is a lack of culturally appropriate assessment tools and empirical research examining neuroception of psychological safety within the Chinese context.

Trauma psychology suggests that the hyperarousal state and risk behaviors observed in patients with mental disorders may not solely represent pathological psychiatric symptoms, but could also reflect defensive behaviors adopted in response to repeated traumatic experiences (5). According to the Polyvagal Theory (PVT), the neuroception of psychological safety encompasses dimensions of somatic sensations, social engagement, and empathic experiences. Neuroception is conceptually distinct from psychological safety and perceived safety, which refer to conscious appraisals of being safe in a given context. According to the Polyvagal Theory, trauma may be associated with altered neuroceptive processing of safety and threat. This theoretical perspective provides a possible framework for understanding the observed associations among traumatic experiences, neuroception of psychological safety, and aggressive behavior. However, the temporal and causal relationships among these variables require longitudinal investigation. This indicates that patients’ perception of safety should incorporate both physical and psychological attributes. Currently, the most established instrument for measuring psychological safety in China is the Security Questionnaire developed by Cong Zhong et al. (6). This scale primarily assesses conscious psychological safety, and its application among patients with mental disorders remains limited. Furthermore, the scale was originally constructed based on initial conceptualizations of psychological safety and semi-structured interviews. Although items pertaining to “physical safety” were initially considered during development, they were ultimately excluded because they failed to meet the required standards in subsequent reliability and validity testing. As a result, it does not capture the neuroceptive, bodily dimension of safety as defined by PVT. The Neuroception of Psychological Safety Scale (NPSS) (7), developed by Morton based on the Polyvagal Theory, addresses this limitation by offering comprehensive dimensional coverage that captures the somatic aspect of safety perception, thereby compensating for the shortcomings of traditional scales.

Our research team has completed the translation and cultural adaptation of the Chinese version of the NPSS, and has verified its reliability and validity. The findings confirm that the scale’s item wording and dimensional structure are culturally appropriate for the Chinese context and clinically applicable. Building on this work, the present study used a cross-sectional design to investigate the following research questions among inpatients with mental disorders: First, to describe the current level of neuroception of psychological safety and the characteristics of its dimensional scores in this population; second, to examine factors associated with patients’ neuroception of psychological safety, including traumatic experiences and psychiatric symptoms; and third, to construct a structural equation model based on the Polyvagal Theory to examine whether the observed associations among trauma, neuroception of psychological safety, and aggression are consistent with a statistical mediation model. The innovation of this study lies in its departure from the traditional “healthcare provider perspective” in psychiatric violence management, instead focusing on the neuroceptive dimension of patients’ neuroception of psychological safety. By utilizing a culturally adapted and validated quantitative assessment tool, this study examines the pattern of associations among trauma, neuroception of psychological safety, and aggressive behaviors, including whether these associations are consistent with a statistical mediation model. The findings of this study provide empirical evidence relevant to understanding aggression risk in psychiatric clinical settings and promote the standardized application of neuroception of psychological safety assessment within the mental health field in China.

## 2 Methods

### 2.1 Study participants

From July to October 2023, eligible patients were recruited from nine wards of a district-level specialized hospital in Shanghai. A stratified sampling approach was applied sequentially by gender and age. Specifically, participants were first divided into eight strata according to sex (male, female) and age groups (<40, 40–49, 50–59, and ≥60 years). The sample size for each stratum was determined based on its proportional distribution in the population. Subsequently, patients within each stratum were randomly selected using the random number function in Excel.

#### 2.1.1 Inclusion criteria and exclusion criteria

The inclusion criteria were as follows: (1) patients diagnosed with a mental disorder according to the ICD-10 criteria, as confirmed by a clinician; (2) aged between 16 and 65 years; (3) conscious, emotionally stable, and possessing adequate communication skills to complete the interview; (4) hospitalized for more than 72 h; (5) deemed by their attending physician to have the cognitive capacity to effectively participate in the assessment; and (6) both patients and their legal guardians provided written informed consent. Patients with a history of alcohol or substance abuse, or those with severe physical illnesses, were excluded. This study was approved by the ethics committee of the authors’ institution (Approval Number: PDJWLL2022038).

#### 2.1.2 Sample size estimation

Based on the requirements for factor analysis (5–10 times the number of independent variables) and the stability criteria for structural equation modeling, with an allowance of 10–20% for invalid responses, the target sample size was determined to be at least 200 participants (8, 9). A total of 230 questionnaires were collected. After excluding 14 questionnaires due to missing data, incorrect completion, patterned responses, or wave-like answering, the final sample comprised 216 participants.

### 2.2 Instruments

#### 2.2.1 General demographic questionnaire

A self-designed general demographic questionnaire was developed through discussions among the research team. The collected information included age, gender, educational level, marital status, type of mental disorder, health insurance coverage, medication, family history of mental illness, frequency of family visits, and family history of mental disorders.

#### 2.2.2 Chinese version of the Neuroception of Psychological Safety Scale (NPSS)

The scale was originally developed by Morton to assess patients’ neuroception of psychological safety over the past month (7). The Chinese version of the Neuroception of Psychological Safety Scale (NPSS) was used to assess participants’ psychological safety over the past month. This version was rigorously translated and culturally adapted following standard guidelines, and its psychometric properties—including the three-factor structure (compassion, social engagement, and bodily sensations), measurement invariance, and criterion validity—have been previously validated in an independent Chinese sample (10). In the present study, the scale demonstrated excellent internal consistency, with a Cronbach’s α coefficient of 0.955 for the total scale and subscale coefficients ranging from 0.861 to 0.938.

#### 2.2.3 Childhood Trauma Questionnaire (CTQ)

The Childhood Trauma Questionnaire, originally developed by Bernstein et al. (11) in its short form, was revised into Chinese by Zhao Xingfu et al. (12). This instrument is used to assess participants’ experiences of childhood maltreatment and neglect. It comprises five dimensions: emotional abuse, physical abuse, sexual abuse, emotional neglect, and physical neglect. Each dimension contains five items, with an additional three items included for validity verification. The scale consists of 28 items in total, each rated on a 5-point scale ranging from 1 (never) to 5 (always). Higher scores indicate more severe experiences of childhood trauma. The Cronbach’s α coefficient was 0.910 in this study.

#### 2.2.4 Modified Overt Aggression Scale (MOAS)

The Modified Overt Aggression Scale, originally developed by Knoedler (13), is an instrument designed to objectively assess the type and severity of aggressive behaviors in patients with mental disorders. MOAS data were derived from the current hospitalization episode and were based on medical record reviews. The assessment period covers the past month. The scale consists of four subscales: verbal aggression, aggression against property, auto-aggression (self-harm), and physical aggression. The MOAS includes four subscales (e.g., verbal aggression, aggression against property) and employs a weighted scoring method, with higher total scores indicating greater aggressiveness. Patients were classified into aggressive behavior groups based on established criteria (14). The Cronbach’s α coefficient of the MOAS was 0.802 in this study.

#### 2.2.5 Positive Symptoms Subscale

The Positive Symptoms Subscale is derived from the Positive and Negative Syndrome Scale (PANSS) (15). It consists of 7 items, each rated on a 7-point scale based on increasing levels of psychopathology. Assessments are conducted by psychiatrists, with higher scores indicating more severe positive symptoms. This subscale has demonstrated good reliability and validity in populations with mental disorders, with a Cronbach’s α coefficient of 0.863 in this study.

#### 2.2.6 Psychiatric Experiences Questionnaire (PEQ)

Various traumatic and harmful experiences that may occur in psychiatric settings were assessed (16). The PEQ consisted of 29 events that could happen during the current hospitalization (e.g., being handcuffed and transported by police van), and the frequency of occurrence was asked. Data collection was conducted by cross-verifying medical record reviews with patient reports. The original questionnaire was in English and was introduced to Hong Kong by KK Wu et al. (17). In this study, the Cantonese expressions from the Hong Kong version were converted into Mandarin, and content clarity and cultural appropriateness were confirmed through cognitive interviews with seven experts and ten patients. After expert review, the item-level content validity index ranged from 0.86 to 1, and the scale-level content validity index was 0.99. We assessed the agreement between patient self-reports and medical record documentation (random subset of 30 individuals). The mean agreement rate was 92.1%, and the mean Kappa was 0.81, indicating excellent reliability (18). This instrument was a formative indicator (each event was relatively independent); therefore, Cronbach’s α was not applicable (19). Its reliability was mainly supported by content validity and cross-cultural validation in Hong Kong.

### 2.3 Data collection

Data collection was conducted between July and October 2023 at a psychiatric hospital in Shanghai. Four investigators who had received unified training and passed competency assessments administered the survey. With the support of hospital administration, the investigators explained the content and purpose of the study to potential participants. Written informed consent was obtained from all patients prior to participation. Due to the impracticality of using mobile phones in the closed ward setting, paper-based questionnaires were administered. All questionnaires were completed within 20 min. Upon completion, the questionnaires were checked on-site for completeness and any omissions or errors were corrected promptly. The collected data were then entered into a computer database in a timely manner.

### 2.4 Statistical analysis

Data were entered and verified using EpiData 3.1 through double-entry by two researchers. Statistical analyses were performed using SPSS 26.0, including descriptive statistics, univariate analysis, Pearson correlation analysis, and multiple linear stepwise regression. Given that the factors associated with patients’ neuroception of psychological safety have not yet been systematically established, and the relationships among potential predictors remain insufficiently supported by empirical evidence, we first employed a stepwise regression procedure (entry criterion α ≤ 0.05, removal criterion α ≥ 0.10) as an exploratory tool for variable screening. However, given the well-recognized limitations of stepwise regression—particularly model instability and inflated Type I error—and to rigorously test the robustness of our findings, we additionally prespecified a theoretically driven forced-entry (Enter) regression model as a sensitivity analysis, simultaneously incorporating clinically important confounders and core study variables into the equation.

To assess the potential threat of common method bias, we conducted the Unmeasured Latent Method Construct (ULMC) analysis. Since our structural equation model employed dimension-level aggregates (item parceling) as observed indicators, for which the Harman single-factor test is not appropriate, we adopted ULMC as a more rigorous alternative (20). Specifically, we added a common method factor to the original four-factor confirmatory factor analysis model, loading all observed variables onto this factor while constraining it to be uncorrelated with the theoretical trait factors. Model fit comparisons (△CFI and △RMSEA) were used to evaluate the severity of common method bias, with △CFI < 0.01 and △RMSEA < 0.015 indicating no significant threat (21).

A structural equation model was constructed using AMOS 23.0 with parameters estimated by the maximum likelihood method. The bootstrap method with 2,000 resamples was used to test indirect effects. A *p*-value < 0.05 was considered statistically significant.

## 3 Results

### 3.1 Descriptive analysis of neuroception of psychological safety in inpatients with mental disorders

#### 3.1.1 General characteristics of the participants

A total of 216 participants were included. The demographic and clinical characteristics of the participants are presented in Table 1, with schizophrenia as the main diagnosis (84.72%) and unmarried patients in the majority (70.83%).

**Table 1:** Demographic and clinical characteristics of the participants (*N* = 216).

Characteristic	Category	*n* (%)/Mean (SD)
Gender	Male	88 (40.74)
	Female	128 (59.26)
Age (years)	18 ~ 29	33 (15.28)
	30 ~ 41	50 (23.15)
	42 ~ 53	81 (37.50)
	54 ~ 65	52 (24.07)
Ethnicity	Han ethnicity	214 (99.07)
	Other	2 (0.93)
Current hospitalization duration (months)		26.57 (12.80)
Family history of mental illness	Yes	66 (30.56)
	No	150 (69.44)
Education level	Primary school	20 (9.26)
	Junior high school	99 (45.83)
	Senior high school	77 (35.65)
	College degree or above	20 (9.26)
Marital status	Unmarried	153 (70.83)
	Married	43 (19.9)
	Divorced	17 (7.9)
	Other	3 (1.4)
Chlorpromazine equivalent dose (mg/day)		444.84 (154.87)
Frequency of family visits	≤ Once every 2 weeks	53 (24.54)
	> Once every 2 weeks to ≤ Once a month	68 (31.48)
	≥ Once a month to ≤ Once every 3 months	61 (28.24)
	> once every 3 months	34 (15.74)
Type of mental disorder	Schizophrenia	183 (84.72)
	Affective disorder	33 (15.27)

#### 3.1.2 Neuroception of psychological safety scores in inpatients with mental disorders

Normality testing indicated that the neuroception of psychological safety scores were approximately normally distributed. The total mean score for neuroception of psychological safety was 3.80 (0.56). The dimension scores, ranked from highest to lowest, were as follows: bodily sensations, 4.00 (0.51); social engagement, 3.73 (0.69); and compassion, 3.72 (0.62).

#### 3.1.3 Patients’ traumatic experiences and psychiatric symptom scores

##### 3.1.3.1 Treatment-related traumatic experiences

The self-reported incidence rate of traumatic events among patients was 90.3%, with 38% of patients experiencing more than four traumatic events. The five most frequently reported events were: being forced to take medication against one’s will (14.17%), witnessing other patients being “taken away” (11.19%), being placed in seclusion or physical restraint (9.05%), lack of privacy during bathing/dressing/toileting (8.21%), and being “removed” by police or psychiatric staff (7.62%).

##### 3.1.3.2 Childhood trauma and psychiatric symptom scores

The dimension scores of childhood trauma were approximately normally distributed, with absolute values of skewness <3 and kurtosis <10. Examination of the scatter plots revealed a linear trend between each pair of variables, indicating that the assumption of bivariate normality was satisfied. Among the dimensions, emotional neglect received the highest score, at 12.75 (2.11), while sexual abuse received the lowest score, at 6.13 (1.28). The mean score on the Positive Symptoms Subscale was 13.56 (4.07), and the mean score on the Psychiatric Experiences Questionnaire was 10.00 (4.56). Detailed results are presented in Table 2.

**Table 2:** Patients’ scores on childhood trauma, positive symptoms, and treatment-related trauma measures (*N* = 216).

Measure	Number of items	Mean (SD)	Kurtosis	Skewness
Childhood Trauma Questionnaire (CTQ)				
Emotional abuse	5	8.77 (2.75)	1.10	1.40
Physical abuse	5	6.48 (1.26)	0.23	−1.24
Sexual abuse	5	6.13 (1.28)	0.78	−0.63
Emotional neglect	5	12.75 (2.11)	−0.10	−1.21
Positive Symptoms Subscale (PANSS)	7	13.56 (4.07)	1.00	0.42
Psychiatric Experiences Questionnaire (PEQ)	29	10 (4.56)	−0.53	−0.09

#### 3.1.4 Levels of neuroception of psychological safety and analysis of associated factors

Univariate analysis revealed statistically significant differences in neuroception of psychological safety scores among patients based on gender, educational level, frequency of family visits, and presence of a family history of mental illness (all *p* < 0.05). Specifically, patients who were male, had higher educational levels, and received more frequent family visits reported higher levels of neuroception of psychological safety.

Pearson correlation analysis showed that the total neuroception of psychological safety score and its dimension scores were significantly negatively correlated with psychiatric treatment-related experiences, all dimensions of childhood trauma, and positive symptom scores (r ranging from −0.212 to −0.688, all *p* < 0.01). Among these, the strongest negative correlation was observed between positive symptoms and the social engagement dimension (*r* = −0.688). Detailed results are presented in Table 3.

**Table 3:** Correlations between neuroception of psychological safety and study variables (*N* = 216).

Variable	Social engagement	Compassion	Bodily safety	NPSS
Psychiatric treatment experiences	−0.440^**^	−0.255^**^	−0.312^**^	−0.406^**^
Childhood trauma (total)	−0.535^**^	−0.316^**^	−0.497^**^	−0.525^**^
Emotional abuse	−0.516^**^	−0.278^**^	−0.42^**^	−0.490^**^
Physical abuse	−0.494^**^	−0.365^**^	−0.434^**^	−0.498^**^
Sexual abuse	−0.488^**^	−0.301^**^	−0.464^**^	−0.485^**^
Emotional neglect	−0.451^**^	−0.307^**^	−0.463^**^	−0.464^**^
Physical neglect	−0.448^**^	−0.212^**^	−0.428^**^	−0.428^**^
Positive symptoms	−0.688^**^	−0.491^**^	−0.503^**^	−0.663^**^

**p < 0.01; NPSS, Neuroception of Psychological Safety Scale.

Multiple linear stepwise regression analysis was performed with the total neuroception of psychological safety score as the dependent variable. Variables that demonstrated statistical significance in univariate analysis and correlation analysis were entered into the model. The coding methods for the independent variables were specified as follows: gender: male = 1, female = 2; educational level: primary school = 1, junior high school = 2, senior high school = 3, college degree or above = 4; positive symptoms: raw value; frequency of family visits: ≤ once every 2 weeks = 1, > once every 2 weeks to ≤ once a month = 2, ≥ once a month to ≤ once every 3 months = 3, > once every 3 months = 4; family history of mental illness: no = 0, yes = 1; childhood trauma: raw value; psychiatric treatment experiences: raw value;duration of illness: raw value. Residual analysis confirmed that the residuals followed a normal distribution (*p* > 0.05) and exhibited homogeneity of variance. The Durbin-Watson statistic was 1.950, indicating that the observations were independent of one another. The results revealed that gender, positive symptoms, childhood trauma, frequency of family visits, educational level, family history of mental illness, and psychiatric treatment experiences were associated with neuroception of psychological safety (all *p* < 0.05). The regression model demonstrated good fit, with an adjusted R^2^ of 0.581 (*F* = 43.652, *p* < 0.001). Tolerance values for the independent variables ranged from 0.691 to 0.985, and variance inflation factor (VIF) values ranged from 1.015 to 1.448, indicating no multicollinearity among the variables (15). The regression equation was as follows: Neuroception of Psychological Safety = 154.538–3.75 × Gender - 1.958 × Positive Symptoms - 0.405 × Childhood Trauma +1.855 × Frequency of Family Visits + 2.962 × Educational Level - 4.122 × Family History of Mental Illness - 0.71 × Psychiatric Treatment Experiences. Detailed results are presented in Table 4.

**Table 4:** Multiple linear regression analysis of factors associated with Neuroception of Psychological Safety (*N* = 216).

Variable	*B*	Standard error	Beta	*t*	*p*	Tolerance	VIF
Constant	154.538	5.542	-	27.885	<0.001	-	-
Gender	−3.756	1.517	−0.113	−2.476	0.014	0.931	1.075
Positive symptoms	−1.958	0.205	−0.488	−9.574	<0.001	0.75	1.333
Childhood trauma	−0.405	0.1	−0.216	−4.064	<0.001	0.691	1.448
Frequency of family visits	1.855	0.758	0.116	2.448	0.015	0.871	1.148
Educational level	2.962	0.882	0.149	3.358	0.001	0.985	1.015
Family history of mental illness	−4.122	1.628	−0.117	−2.531	0.012	0.919	1.088
Psychiatric treatment experiences	−0.71	0.337	−0.106	−2.104	0.037	0.768	1.302

B = unstandardized coefficient; Beta = standardized coefficient; VIF = variance inflation factor. F = 43.652, R^2^ = 0.595, adjusted R^2^ = 0.581.

To further evaluate the robustness of the findings against the potential limitations of stepwise regression (e.g., model instability and inflated Type I error), we conducted a sensitivity analysis using a theoretically driven forced-entry (Enter) regression model. In this model, gender, age, positive symptoms, and childhood trauma were simultaneously forced into the regression equation as predefined covariates based on clinical relevance. The results demonstrated that positive symptoms (*B* = −2.168, *β* = −0.540, *p* < 0.001) and childhood trauma (*B* = −0.459, *β* = −0.244, *p* < 0.001) remained statistically significant, with effect sizes and directions highly consistent with those from the stepwise model. Gender also remained significant (*B* = −5.309, *β* = −0.160, *p* = 0.001), whereas age was not significantly associated with neuroception of psychological safety (*B* = 0.051, *β* = 0.038, *p* = 0.428). The high concordance between the two modeling approaches supports the stability of our primary conclusions, indicating that the observed associations are unlikely to be artifacts of stepwise variable selection.

### 3.2 Mediating role of neuroception of psychological safety

#### 3.2.1 Correlation analysis among variables

Preliminary correlation analysis conducted prior to testing the mediating effect revealed that both childhood trauma and treatment-related trauma were significantly positively correlated with aggressive behaviors (*p* < 0.01) and significantly negatively correlated with neuroception of psychological safety (*p* < 0.01). Furthermore, neuroception of psychological safety was significantly negatively correlated with aggressive behaviors (*p* < 0.01). These findings met the prerequisite conditions for conducting mediation analysis. Detailed results are presented in Table 5.

**Table 5:** Correlations among study variables (*N* = 216).

Variable	Neuroception of psychological safety	Aggressive behavior	Childhood trauma	Psychiatric treatment experiences
Neuroception of psychological safety	1			
Aggressive behavior	−0.485^**^	1		
Childhood trauma	−0.525^**^	0.540^**^	1	
Psychiatric treatment experiences	−0.406^**^	0.437^**^	0.398^**^	1

^**^p < 0.01.

#### 3.2.2 Structural equation model fit and path analysis

Based on the Polyvagal Theory and the clinical hypothesis model (Figure 1), childhood trauma was specified as an exogenous latent variable, with its observed indicators being the scores of the five subscales (emotional abuse, physical abuse, sexual abuse, emotional neglect, and physical neglect) of the Childhood Trauma Questionnaire (CTQ). Psychological safety was specified as an endogenous latent variable, with its observed indicators being the scores of the three dimensions (social engagement, compassion, and bodily sensations) of the NPSS. Aggressive behavior was included as the outcome variable, with its observed indicators being the scores of the four subscales (verbal aggression, aggression against property, autoaggression, and physical aggression against others) of the Modified Overt Aggression Scale (MOAS). Experiences related to psychiatric treatment, having only a single observed indicator (number of treatment episodes or whether treated), were included in the model as a manifest variable. The standardized factor loadings of the observed indicators ranged from 0.59 to 0.83 (all *p* < 0.001), indicating that the measurement model was adequate. All model fit indices met the recommended criteria: *χ^2^*/df = 2.185 (< 3), RMSEA = 0.074 (< 0.08), IFI = 0.964, TLI = 0.952, CFI = 0.963 (all > 0.95), PGFI = 0.601, and PNFI = 0.719 (both > 0.5). These results indicated that the model structure demonstrated a good fit with the observed data.

**Figure 1:**
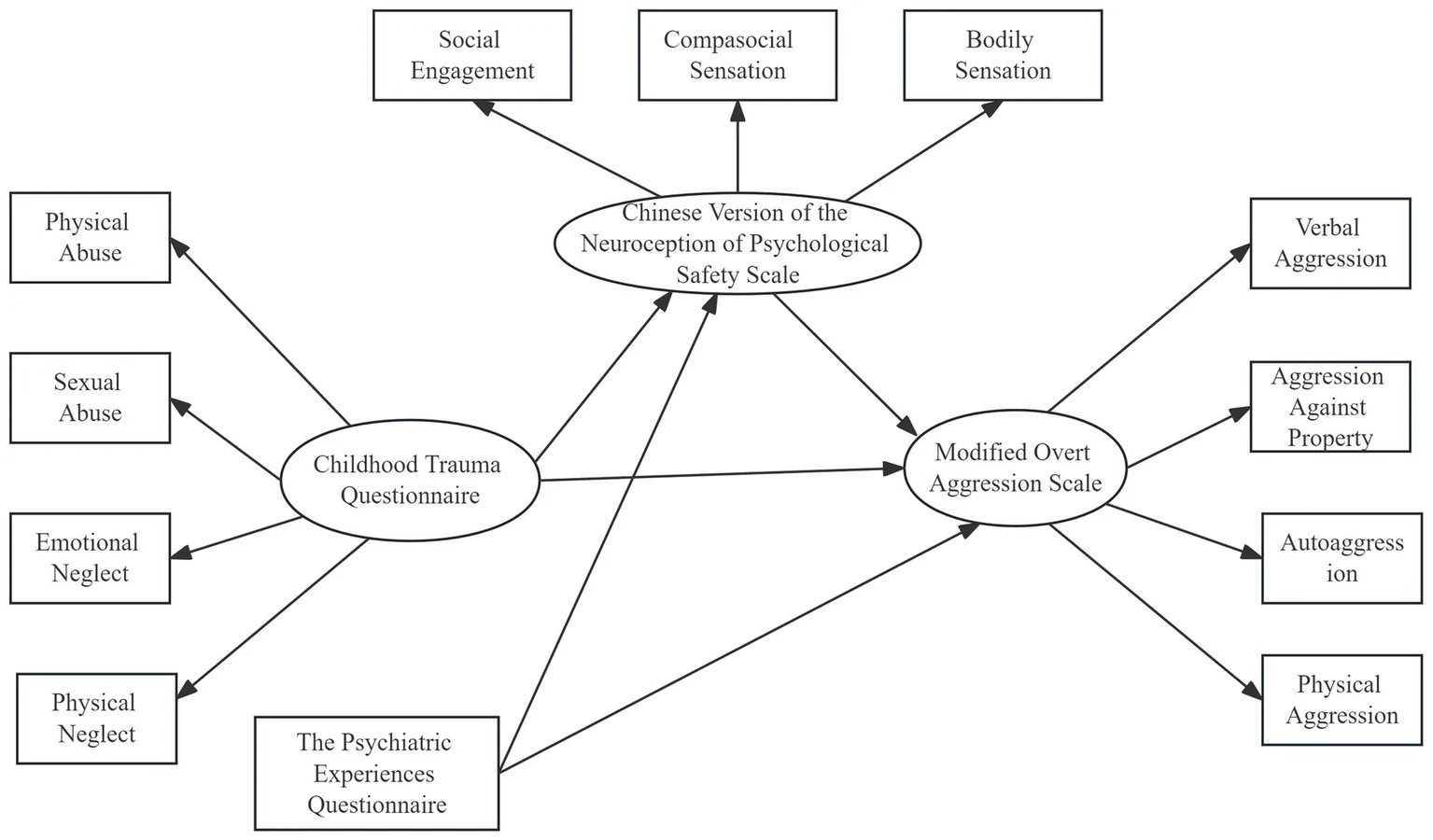
Postulated mediational path model.

To assess common method bias, we conducted the Unmeasured Latent Method Construct (ULMC) analysis. A common method factor was added to the four-factor model, with all observed indicators loading onto it while constraining it to be uncorrelated with the trait factors. Model comparison showed that adding the method factor improved model fit (Model B: χ^2^/df = 1.614, RMSEA = 0.053, CFI = 0.985; △CFI = 0.022, △RMSEA = −0.021). The ΔCFI value of 0.022 exceeds the recommended 0.01 threshold (21), suggesting the possible presence of common method variance. However, this concern is partially mitigated by the objective extraction of the outcome variable (MOAS) from medical records, whereas the predictors were self-reported. Nonetheless, the potential influence of common method bias cannot be entirely ruled out.

Path analysis results (Table 6) revealed the following:

1. Both childhood trauma and trauma related to psychiatric treatment were significantly and negatively associated with neuroception of psychological safety (*β* = −0.471, *p* < 0.001; *β* = −0.230, *p* < 0.001, respectively), indicating a strong negative correlation between these two types of traumatic experiences and the level of neuroception of psychological safety among hospitalized patients with mental disorders.
2. Neuroception of psychological safety was significantly and negatively correlated with aggressive behavior (*β* = −0.240, *p* < 0.01), suggesting that patients with higher levels of neuroception of psychological safety reported lower levels of aggressive behavior. Thus, neuroception of psychological safety may represent a clinically relevant correlate of lower aggression.
3. Childhood trauma (*β* = 0.353, *p* < 0.001) and trauma related to psychiatric treatment (*β* = 0.215, *p* < 0.01) were each positively and significantly associated with aggressive behavior. Furthermore, both types of trauma were indirectly related to aggressive behavior through their negative association with neuroception of psychological safety, suggesting a significant indirect association through neuroception of psychological safety, consistent with a partial statistical mediation model.

**Table 6:** Path analysis results of the structural equation model (*N* = 216).

Path relationship	Estimate (*β*)	Standard error (*S. E.*)	Critical ratio (*C. R.*)	*p*
Neuroception of psychological safety ← Psychiatric treatment experiences	−0.230	0.236	−3.419	<0.001
Neuroception of psychological safety ← Childhood trauma	−0.471	0.234	−6.647	<0.001
Aggressive behavior ← Neuroception of psychological safety	−0.240	0.010	−2.942	0.003
Aggressive behavior ← Childhood trauma	0.353	0.032	4.518	<0.001
Aggressive behavior ← Psychiatric treatment experiences	0.215	0.029	3.223	0.001

### 3.3 Mediation analysis

To evaluate the indirect effects within the mediating effect, the bootstrap method was employed with 2,000 bootstrap resamples and a 95% confidence interval, and the sampling method used was the bias-corrected nonparametric percentile approach. The bootstrap analysis identified significant indirect associations through neuroception of psychological safety for both childhood trauma and psychiatric treatment-related trauma in relation to aggressive behavior. These findings were consistent with partial statistical mediation; however, given the cross-sectional design, they should not be interpreted as evidence of causal mediation.

1. For the relationship between childhood trauma and aggressive behavior: the total effect was 0.466 (95% *CI* [0.314, 0.612]), the direct effect was 0.353, accounting for 75.8% of the total effect (95% *CI* [0.165, 0.547]), and the indirect effect was 0.113, accounting for 24.2% of the total effect (95% *CI* [0.046, 0.219]).
2. For the relationship between psychiatric treatment experiences and aggressive behavior: the total effect was 0.270 (95% *CI* [0.121, 0.427]), the direct effect was 0.215, accounting for 79.6% of the total effect (95% *CI* [0.082, 0.345]), and the indirect effect was 0.055, accounting for 20.4% of the total effect (95% *CI* [0.002, 0.165]). Detailed results are presented in Table 7.

**Table 7:** Mediating effect of neuroception of psychological safety (*N* = 216).

Path	Type of effect	Effect value	Standard error	95% *CI*	*p*	Proportion of effect
Childhood trauma → Aggressive behavior	Total effect	0.466	0.078	[0.314,0.612]	0.001	100%
	Direct effect	0.353	0.097	[0.165,0.547]	0.002	75.8%
	Indirect effect	0.113	0.045	[0.046,0.219]	0.007	24.2%
Psychiatric treatment experiences → Aggressive behavior	Total effect	0.270	0.079	[0.121,0.427]	0.001	100%
	Direct effect	0.215	0.067	[0.082,0.345]	0.001	79.6%
	Indirect effect	0.055	0.040	[0.002,0.165]	0.036	20.4%

To evaluate the potential impact of unmeasured confounding, we calculated the E-value for the total effect using the correlation coefficient method (22). The E-value for childhood trauma was 2.74, indicating that the finding is relatively robust to unmeasured confounding. The E-value for psychiatric treatment-related experiences was 1.74, suggesting that this association is more sensitive to unmeasured confounding (23). These E-value analyses provide information regarding the potential robustness of the observed associations to unmeasured confounding; however, they do not overcome the temporal limitations of the cross-sectional design or establish causality.

## 4 Discussion

Grounded in the Polyvagal Theory, this study focused on inpatients with mental disorders and systematically described the current status and dimensional characteristics of their neuroception of psychological safety. Factors associated with neuroception of psychological safety were identified, and significant indirect associations through neuroception of psychological safety were observed between traumatic experiences and aggressive behaviors. These findings were consistent with a partial statistical mediation model. These findings suggest that neuroception of psychological safety may be clinically relevant to aggression and provide a basis for future longitudinal and interventional studies examining whether enhancing psychological safety is associated with reductions in aggression risk. The findings also provide additional support for the clinical applicability of the Chinese version of the NPSS in this population.

### 4.1 Current status of neuroception of psychological safety in inpatients with mental disorders

The results of this study revealed that the total score of neuroception of psychological safety among inpatients with mental disorders was 3.80 (0.56). The dimension scores, ranked from highest to lowest, were bodily sensations, social engagement, and compassion. This ranking differs from the English version of the NPSS in healthy populations (social engagement> compassion > bodily sensations) (7). This discrepancy may reflect differences in cultural background, population characteristics, and clinical context.

From a dimensional perspective, the bodily sensations dimension received the highest score. This relatively higher score may be associated with the structured safety protocols and standardized treatment procedures implemented in the closed inpatient setting.

In contrast to the bodily sensations dimension, scores on the social engagement and compassion dimensions were relatively low. This pattern suggests relatively lower perceived social connectedness and empathic experience in this inpatient sample. These findings may be related to the restricted scope of social interaction resulting from the closed ward management, as well as the impairments in cognitive and affective functions associated with the illness itself, which may further diminish patients’ willingness to engage socially and their capacity for empathic experience.

### 4.2 Factors associated with neuroception of psychological safety in inpatients with mental disorders

#### 4.2.1 Associations of demographic and clinical support factors with neuroception of psychological safety

The results identified gender, educational level, family history of mental illness, and frequency of family visits as factors independently associated with neuroception of psychological safety. Male patients reported significantly higher neuroception of psychological safety than females, consistent with previous findings (24, 25). This finding may be related to differences in post-discharge social concerns and emotional responses reported in previous studies (26). Higher educational levels were associated with greater neuroception of psychological safety, the association with educational level may be related to differences in information processing and coping abilities (27). Conversely, a family history of mental illness was linked to lower neuroception of psychological safety, the association with family history may reflect differences in illness-related experiences or concerns about recurrence. Frequency of family visits was positively correlated with neuroception of psychological safety, highlighting the role of family emotional support as a vital link to the outside world in closed inpatient settings (28). Sensitivity analysis further confirmed the robustness of the gender difference (*B* = −5.309, *β* = −0.160, *p* = 0.001), whereas age was not significantly associated with neuroception of psychological safety (*B* = 0.051, *β* = 0.038, *p* = 0.428), suggesting that clinical and psychosocial factors may outweigh age-related influences.

#### 4.2.2 Negative associations of traumatic experiences and psychiatric symptoms with neuroception of psychological safety

Stepwise regression analysis revealed that childhood trauma, psychiatric treatment-related trauma, and positive symptoms were significantly negatively associated with neuroception of psychological safety. To test the robustness of the core findings, a forced-entry sensitivity analysis (including gender, age, positive symptoms, and childhood trauma) was conducted, which confirmed that positive symptoms (*B* = −2.168, *β* = −0.540, *p* < 0.001) and childhood trauma (*B* = −0.459, *β* = −0.244, *p* < 0.001) remained stable across both models, supporting the robustness of these associations.

The mean score for childhood trauma in this study was 44.72 (8.70), which is consistent with findings reported by Chen et al. (29). This suggests that higher levels of childhood trauma were associated with lower neuroception of psychological safety. Within the stressful context of hospitalization, previous traumatic memories may be reactivated, which may be associated with feelings of insecurity.

Psychiatric treatment-related trauma was also significantly negatively associated with neuroception of psychological safety. In this study, 90.3% of patients reported experiencing treatment-related traumatic events, with the five most frequently reported incidents all associated with involuntary treatment measures, such as being forced to take medication against one’s will and witnessing another patient being “taken away.” These findings are consistent with reports from Hong Kong and the United States (15, 16). Such traumatic events are negatively associated with patients‘sense of autonomy and dignity, and correlate with lower trust in the treatment environment and lower neuroception of psychological safety. From the perspective of Polyvagal Theory, these experiences may be associated with reactivation of traumatic memories and heightened autonomic vigilance, which may reflect lower perceived psychological safety. This interpretation remains theoretical and requires longitudinal validation. These findings suggest that humanistic safety management informed by Trauma-Informed Care (TIC) principles may warrant greater attention in future psychiatric nursing research and practice.

The severity of positive symptoms was also negatively associated with neuroception of psychological safety. Positive symptoms such as hallucinations and delusions heighten patients’ vigilance toward their surroundings. In particular, persecutory delusions may exacerbate hostile attribution bias (30), keeping patients in a prolonged state of tension and fear, thereby diminishing their neuroception of psychological safety. Notably, positive symptoms showed the strongest and most stable association across both models, suggesting that their relationship with neuroception of psychological safety warrants further investigation in longitudinal studies.

### 4.3 Mediating role of neuroception of psychological safety in the relationship between traumatic experiences and aggressive behavior

The structural equation model constructed based on the Polyvagal Theory demonstrated a good fit with the data. The results revealed significant indirect associations through neuroception of psychological safety in the relationships of both childhood trauma and psychiatric treatment-related trauma with aggressive behaviors, accounting for 24.2 and 20.4% of the total associations, respectively. These findings are compatible with the Polyvagal Theory, which provides one possible theoretical interpretation of the observed associations among trauma, neuroception of psychological safety, and aggression (31). However, the present cross-sectional data cannot determine the temporal ordering of these variables or establish the proposed neuroceptive mechanism. Longitudinal studies incorporating repeated measurements are needed to examine whether changes in neuroception precede changes in aggressive behavior.

Both childhood trauma and treatment-related trauma not only demonstrated direct positive associations with aggressive behavior but were also negatively correlated with neuroception of psychological safety, and significant indirect associations with aggressive behavior through neuroception of psychological safety were also observed. From a theoretical perspective, the Polyvagal Theory suggests that traumatic experiences may be associated with prolonged hypervigilance. Although this mechanism is theoretically plausible, the present cross-sectional findings cannot establish this temporal or causal sequence. In the absence of neuroception of psychological safety, patients may be more prone to interpreting neutral events as threatening stimuli, thereby exhibiting defensive behaviors such as verbal or physical aggression. This mechanism is hypothesized to be consistent with the findings of Wang et al. (32).

Neuroception of psychological safety is negatively associated with aggressive behavior, suggesting that this construct may be clinically relevant. Thus, its potential role in aggression prevention warrants further investigation in future intervention studies. Internationally, trauma-informed care guidelines (Substance Abuse and Mental Health Services Administration, 2014) have been adopted, and trauma-informed group psychological interventions implemented with inpatients with mental disorders have been shown to enhance patients’ sense of safety, reduce suicidal ideation (33), and alleviate psychological distress (34). The observed association between neuroception of psychological safety and aggression suggests that this construct may be relevant for future clinical research. Existing trauma-informed approaches provide a basis for prospective investigation (35, 36); however, the present study cannot determine whether modifying neuroception of psychological safety would alter aggressive behavior. Future interventional studies are needed to test this possibility directly.

Several limitations should be acknowledged. First, the cross-sectional design precludes temporal or causal inference; therefore, the observed indirect associations should be interpreted as statistical mediation rather than causal mediation. Second, participants were recruited from a single psychiatric hospital in Shanghai, and 84.72% were diagnosed with schizophrenia. In addition, patients with alcohol or substance abuse were excluded. Thus, generalizability to patients with mood disorders, personality disorders, substance use disorders, and community psychiatric populations may be limited. Third, some key variables relied on self-report measures, which may introduce reporting bias. Fourth, our ULMC analysis suggested the possible presence of common method variance, as indicated by a △CFI exceeding the 0.01 threshold. Although the objective measurement of MOAS from medical records partially mitigates this concern, the self-reported nature of childhood trauma and psychological safety remains subject to reporting biases, such as social desirability and recall inaccuracy. Future studies could incorporate clinician-administered trauma interviews or observational measures of psychological safety to further minimize these biases.

Finally, stepwise regression may introduce model-selection bias, and several clinically relevant variables were not fully accounted for in the SEM, including overall illness severity, medication dosage, length of hospitalization, involuntary admission status, and previous history of violence. Although positive symptom severity was assessed, residual confounding cannot be excluded. Future longitudinal and multicenter studies with more diverse samples and prespecified clinical covariates are needed to validate these findings.

## 5 Conclusion

The findings support the applicability and internal consistency of the previously validated Chinese NPSS in this inpatient sample. The findings support the applicability and internal consistency of the previously validated Chinese NPSS in this inpatient sample. Among the three dimensions, social engagement and compassion showed relatively lower scores than bodily sensations.

This study identified several factors independently associated with neuroception of psychological safety. A significant indirect association through neuroception of psychological safety was observed in the relationships of childhood trauma and psychiatric treatment-related trauma with aggressive behavior, consistent with partial statistical mediation. Traumatic experiences were independently associated with aggressive behavior, and a statistically significant indirect association through neuroception of psychological safety was also observed. This suggests that neuroception of psychological safety may partially account for the observed association between trauma and aggression. However, due to the cross-sectional design, future longitudinal studies are needed to validate these findings. However, given the exploratory nature of the stepwise regression approach and the cross-sectional design, these findings should be interpreted with caution and require confirmation in future longitudinal studies using more stable modeling techniques (e.g., LASSO or structural equation modeling).

This study suggests that in psychiatric clinical practice, attention should be paid to patients’ neuroception of psychological safety needs, with a focus on the negative association of both direct and indirect traumatic events, as well as patients’ rights to engage in their own safety. The observed association between neuroception of psychological safety and aggression suggests that psychological safety may be clinically relevant and warrants further prospective investigation. Future longitudinal and interventional studies should examine whether changes in neuroception of psychological safety are accompanied by changes in aggressive behavior. The present cross-sectional study does not establish that interventions targeting psychological safety reduce aggression. The present cross-sectional study does not establish the efficacy of these strategies in reducing aggressive behavior. This study offers a novel perspective for violence management in psychiatric clinical settings and provides an empirical foundation for the standardized application of neuroception of psychological safety assessment.

## Data Availability

The raw data supporting the conclusions of this article will be made available by the authors, without undue reservation.
